# Non Melanoma Skin Cancer Pathogenesis Overview

**DOI:** 10.3390/biomedicines6010006

**Published:** 2018-01-02

**Authors:** Dario Didona, Giovanni Paolino, Ugo Bottoni, Carmen Cantisani

**Affiliations:** 1 Klinik für Dermatologie und Allergologie, Universitätsklinikum Marburg, Baldingerstraße, 35043 Marburg, Germany; didona.dario@gmail.com; 2 Department of Dermatology, Sapienza Università di Roma, Rome 00100, Italy; paolgio@libero.it; 3 Department of Dermatology, Università della Magna Grecia, Catanzaro 88100, Italy; didona.dermatology@gmail.com

**Keywords:** actinic keratosis, pathogenesis, precancerous conditions, skin neoplasms

## Abstract

(1) Background: Non-melanoma skin cancer is the most frequently diagnosed cancer in humans. The process of skin carcinogenesis is still not fully understood. However, several studies have been conducted to better explain the mechanisms that lead to malignancy; (2) Methods: We reviewed the more recent literature about the pathogenesis of non-melanoma skin cancer focusing on basal cell carcinomas, squamous cell carcinoma and actinic keratosis; (3) Results: Several papers reported genetic and molecular alterations leading to non-melanoma skin cancer. Plenty of risk factors are involved in non-melanoma skin cancer pathogenesis, including genetic and molecular alterations, immunosuppression, and ultraviolet radiation; (4) Conclusion: Although skin carcinogenesis is still not fully understood, several papers demonstrated that genetic and molecular alterations are involved in this process. In addition, plenty of non-melanoma skin cancer risk factors are now known, allowing for an effective prevention of non-melanoma skin cancer development. Compared to other papers on the same topic, our review focused on molecular and genetic factors and analyzed in detail several factors involved in non-melanoma skin cancer.

## 1. Introduction

Non-melanoma skin cancer (NMSC) is by far the most frequently diagnosed cancer [1]. The most common NMSC are basal cell carcinoma (BCC) and squamous cell carcinoma (SCC), respectively 70% and 25% of NMSC, although skin cancers could arise from each host cell of the skin [1]. NMSC show different behavior, growth, and metastatic capability, however, both BCC and SCC have a good prognosis, especially when detected at their initial stages [2]. BCC contributes minimally to the NMSC mortality rate (MR). Indeed, metastatic BCC shows an incidence of 1 case per 14,000,000 and 2 patients per 14,000,000 who die from locally advanced BCC. Therefore, a MR of 0.02 per 10,000 is to be expected [1,2]. On the other hand, SCC shows a variable metastatic rate of 0.1–9.9% and it accounts for about 75% of deaths due to NMSC [1,2]. Although the first-choice therapy is still surgical excision, plenty of alternative approaches have been reported to manage NMSC, including photodynamic therapy, cryotherapy, topical imiquimod 5%, and topical diclofenac sodium 3% [2]. Indeed, it is mandatory to obtain remarkable aesthetical results in the case of NMSC-affected areas such as the lips and the face [2]. 

BCC is characterized by cells that resemble epidermal basal cells and it is the least aggressive NMSC [2]. Indeed, BCC shows a low degree of malignancy, despite of the capability of local invasion, tissue destruction, recurrence, and a limited potential for metastasis [2]. Individual risk factors for BCC include gender, age, immunosuppression, genetic diseases (e.g., Gorlin–Goltz syndrome), and Fitzpatrick skin types I and II [2]. However, ultraviolet (UV) radiation plays the most important role in BCC pathogenesis, although the relationship between UV radiation and BCC development remains highly controversial [2]. BCC develops primarily on sun-exposed skin. Indeed, BCC is rarely found on palmoplantar surfaces and never appears on the mucosa [2].

SCC is characterized by atypical proliferation of invasive squamous cells, which could metastatize. In addition, SCC show a considerable potential for recurrence, which depends on the tumor size, degree of histological differentiation, depth of the lesion, perineural invasion, patient’s immune system, and anatomic localization [2]. Several risk factors have been reported in SCC patients, including Fitzpatrick skin types I and II, outdoor occupation, human papillomavirus (HPV) types 16, 18 and 31, and cutaneous genetically inherited skin diseases, like albinism, xeroderma pigmentosum and epidermodysplasia verruciformis [2]. However, the most important risk factor is represented by UV radiation and sunlight [3]. Indeed, a direct correlation between psoralen and UVA (PUVA) exposure and the incidence of SCC has been reported [3]. Usually SCC arises on sun-exposed areas. Indeed, about 55% of all SCC involves the head and neck [1,2]. In addition, SCC frequently occurs on the extensor surfaces of the hands and forearms (18%) [1,2]. Nevertheless, up to 13% of SCC cases arise on the legs [2]. 

Individuals who develop BCC have an elevated risk of developing new foci of BCC, as well as other types of skin cancer, including melanoma and SCC [2]. Their incidence has increased strongly over time, reflecting also our ageing population [1]. Furthermore, the prevalence of BCC and SCC has increased by 35% and 133%, respectively, over the last 20 years [1,3]. Actinic keratoses (AKs), considered as the earliest manifestation of SCC, are extremely common, showing a prevalence greater than 40% in the adult population [1]. According to some authors, AKs affect half of the global population, although the prevalence varies according to geographical location and age [1,2,3,4]. AKs occur usually on chronically light-exposed skin [3]. AKs share several pathological features with SCC, and they represent a continuum in a multistep process over the years on chronically sun exposed fair skin [2]. Normal-appearing skin that surrounds AKs may develop AKs, because of the UV exposure and expression of molecular alteration, including p53 mutations [2]. This whole area is today known as “field cancerization” [2].

## 2. NMSC and AK: Factors Involved in the Pathogenesis

**Figure 1:**
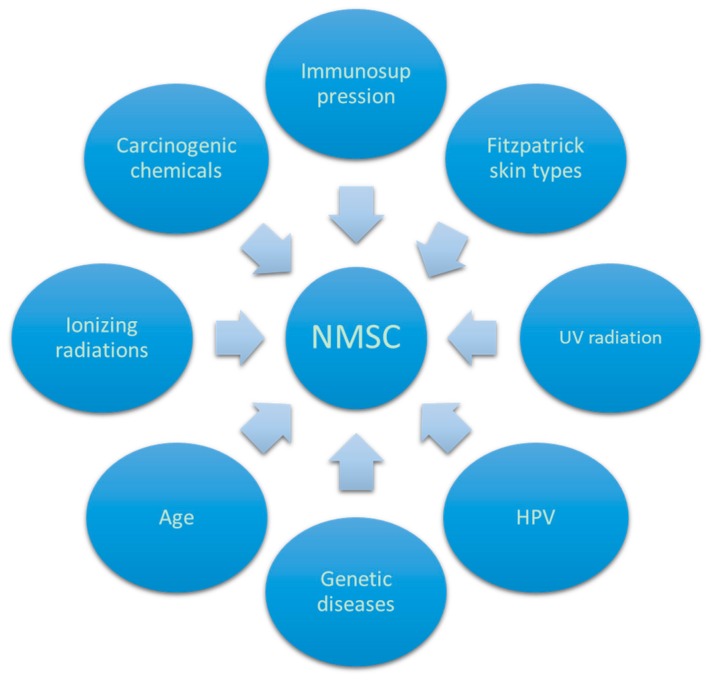
Factors involved in non-melanoma skin cancers (NMSC) pathogenesis. UV: Ultraviolet; HPV: Human papilloma virus.

In the pathogenesis of NMSC and AKs different factors play an important role, including UV rays, X-rays, HPV, arsenic compounds, and other chemical products (Figure 1).

### 2.1. UV Role

As reported by several papers, the primary risk factor for cutaneous carcinogenesis is cumulative UV exposure from sunlight and/or tanning beds, which lead to UV-induced alteration in skin protein expression [2,3,4]. UV exposure is considered as a complete carcinogen, since it affects each stage of carcinogenesis. In fact, it leads to cellular damage because of the reduction of cell-mediated immune responses, production of reactive oxygen species (ROS) and DNA alteration [5,6,7,8]. The earliest event after high UV exposure is keratinocyte apoptosis led by the p53/p21/bax/bcl-2 pathway followed by a hyperproliferative phase, leading to epidermal hyperplasia [5,6]. Chronic exposure to nonionizing solar radiation, specifically UVA and UVB, is the most important risk factor in BCC pathogenesis [2,3,4]. Indeed, UVB-induced carcinogenesis amplifies the risk of developing BCC in immunosuppressed patients and in people with Fitzpatrick skin types I and II [2]. It has been reported that UV irradiation of keratinocytes enhances the pro-opiomelanocortin gene (POMC) and α-melanocyte-stimulating hormone (αMSH) production, which are critically involved in determining whether the skin produces brown-black pigment (eumelanin) or red-yellow pigment (pheomelanin) [2]. Furthermore, it must be highlighted that UV radiation induces not only direct DNA damage but also indirect DNA damage by producing free radicals and UV-induced immunosuppression [3]. Indeed, the Langerhans cells (LCs) are largely affected by UV radiation, which leads to the loss of the dendritic network that they form in the epidermis [3]. In addition, it has been reported that LCs migrate to lymph nodes after UV irradiation, activating natural killer T-cells (NK-T cells) that produce interleukin (IL)-4, which shows immunosuppressive activity [3,7]. In addition, UV-damaged LCs can induce Treg which produce IL-10, another immunosuppressive cytokine [3,7]. It has also been reported that mast cells (MCs) in skin show an increase in response to UV radiation because of IL-33 production from keratinocytes and dermal fibroblasts [3,7]. Consequently, the number of MCs in the B cell areas of the draining lymph nodes increases, stimulating the IL-10-producing B cells, which show a regulatory, immunosuppressive function [7]. 

It has been suggested that exposure to chronic UVB radiation determines heparanase activation, which causes the degradation of heparin sulfate and increments the interaction between the epidermal growth factor and the dermis [9]. Indeed, it has been reported that the cutis consists of hyaluronic acid (HA), dermatan sulfate (DS), heparan sulfate (HS) and keratan sulfate (KS), which play a pivotal role in several processes in the skin, including migration and cell proliferation [10]. Chondroitin sulfate (CS), DS, KS, heparin (HEP) and HS fall in the category of sulfated glycosaminoglycans (GAGs), while hyaluronic acid belongs to the class of non-sulfate GAGs [11]. GAGs affect several biological processes through their interaction with various proteins, including chemokines and cytokines [12]. 

Proteoglycans (PGs) are composed of protein scaffolds and GAGs strains [11,12]. PGs participate in in the organization of collagen fibers, and affect the differentiation and organization of the extracellular matrix [13]. Heparan sulfate proteoglycans (HSPG) play a pivotal role in the extracellular matrix, influencing the integrity of cellular membranes [14]. Heparanase cleaves proteoglycan strains, promoting the growth of the tumor cells, leading to the formation of oligosacharides that enhance angiogenesis and production growth factors, leading in turn to cell proliferation and inflammation [15]. Therefore, heparanase is involved in BCC and SCC formation [15]. Intermittent UV exposure may be most often associated with BCC and melanoma appearance while continuous exposure is associated with SCC.

In addition, UV exposure affects the p53 expression, which is altered in both AKs and SCC [7]. Therefore, this mutation could further confirm that AK is an invasive SCC (iSCC) precursor. Indeed, cutaneous SCC is thought to arise via a multistep process, gradually acquiring mutations that lead to more aggressive behavior [2]. 

### 2.2. X-rays Role

As reported by several papers, X-rays play a role in the pathogenesis of NMSC [16,17,18]. Lichter et al. reported that therapeutic ionizing radiations (IRs), such as X-rays, lead to an increased risk of both BCC and SCC [17]. In particular, radiation therapy for acne has been reported to be associated with about a threefold risk of a new BCC [16]. An increased risk of skin cancer has been observed with occupational, therapeutic, and atomic bomb exposure to IRs [16,17,18]. In addition, it has been reported that the risk of NMSC is higher among those who received radiation therapy at an earlier age [16].

Although studies on atomic bomb survivors have described a relationship only between BCC and ionizing radiations, a recent study reported an odd ratio of 5.7 and 4.8 respectively for BCC and SCC after non-diagnostic radiation exposure [19]. In addition, in patients who received ionizing radiation therapies, a relative risk of 1.7 for new BCC lesions and of 1.0 for SCC lesions has been reported [16]. The latency period between the first exposure and the NMSC development is at least 20 years, although it is difficult to separate the effects of latency from those of age at treatment and type of therapy received [17]. The risk of developing BCC and SCC is confined to the irradiated anatomic area [16,17,18]. Absorption of ionizing radiations leads to a direct breaking of chemical bonds or to the production of radicals that produce massive damage in cellular molecules, including lipids and nucleic acids [19]. Exposure to IRs often leads to exogenous damage, especially to single or double stranded breaks (DSBs). DSBs are DNA alterations that lead to cell death [20]. After DSBs, the histone H2AX moves to the involved area and is converted to the phosphorylate γ-H2AX form in order to repair the damage [21,22]. It has also been reported that the accumulation of p53 after cellular damage could increase cellular mass after IRs exposure [23]. P53 accumulation can lead to cell cycle arrest, DNA repair, or apoptosis [24]. 

IRs are also used as a treatment option for invasive or inoperable BCCs, showing 5 years control rates of 89–100% [2,17]. In addition, radiotherapy represents an important therapeutic strategy against recurrent BCC or morphea-type BCC [17]. However, BCC that recur after radiotherapy are aggressive, invasive and very difficult to eradicate, also showing high recurrence rates after surgical excision [17].

### 2.3. HPV Role

Cutaneous HPV is classified into alpha, beta and gamma types. Beta-HPV is thought to be a cofactor in SCC pathogenesis in immunosuppressed patients. Indeed, many studies have detected DNA from multiple beta-HPV types in SCC lesions, concluding that beta-HPV species 2 is a high-risk subtype [25,26,27]. Beta-papillomaviruses are thought to have an early role in SCC tumorigenesis, altering cell cycle progression, DNA repair, and immune surveillance, leading to clonal expansion of keratinocytes with UVinduced DNA damage [28]. In addition, some alpha-HPV types have also been implicated in SCC [29]. More precisely, HPV77, an alpha-papillomavirus detected in only cutaneous lesions of immunosuppressed patients, contains a p53-DNA binding site. Once activated by UVR, p53 is thought to stimulate HPV77 promoter activity, leading to the production of E6 and E7 proteins that de-regulate p53 and Rb tumor suppressor pathways [29].

However, the exact role of HPV in SCC remains unclear, because HPV DNA has been found also in normal skin samples from SCC patients [30]. It must be highlighted that human leukocyte antigen (HLA) allele groups positively associated with SCC in immunosuppressed patients may encode the HLA protein with reduced efficiency in presenting tumors or HPV antigens, as reported for HLA-DRB1/07 allele. HLA-DRB1/07 allele is associated with impaired presentation of the L1 antigen of HPV8 to CD4+ T lymphocytes, provoking an ineffective Th2-mediated immune response [31].

Genital SCC HPV also plays a role. Indeed, HPV leads to the expression of viral genes E6 and E7, which inactivates tumor suppressor genes [32,33]. High heat shock protein (Hsp)70 levels have been detected in penile SCC. This suggests that it may help tumorous cells to survive apoptosis and necrosis, in part because it was also found to be elevated in several other cancers [33]. In addition, C3 was not detected in penile SCC samples, suggesting that viral proteins are involved in counteracting the host immune response. Therefore, it could be postulated that HPV could provide a favorable milieu for the development of SCC [33].

### 2.4. Carcinogenic Chemicals and Arsenic

The risk of developing SCC is also increased by exposure to carcinogenic chemicals, above all arsenic [34]. Indeed, the expression of several proteins, including keratin 7 and keratin 9, is increased after in vitro arsenic exposure [34]. Contrarily, the production of involucrin is reduced [35]. In C57BL/6-resistant and DBA/2 sensitive mouse models it has been reported that several proteins, including S100, proteins A8 and A9, were elevated after topical application of 12-*O*-tetradecanoylphorbol-13-acetate (TPA), a potent promoter of carcinogenesis [36]. These proteins were related to inflammatory pathways that affect skin neoplastic growth, such as tumor necrosis factor (TNF)-α and nuclear factor (NF)-κB [36]. In addition, it has been demonstrated that CD151, a member of the transmembrane 4 superfamily, induced skin chemical carcinogenesis and promoted the development of SCC, because CD151 might induce the activator of transcription 3 (STAT3) [37]. Therefore, it has been concluded that chemically induced carcinogenesis in animal models may be promoted by inflammation [36]. 

### 2.5. Immunosuppression

Immunosuppression also plays a role in carcinogenesis, leading to an easier NMSC development. Indeed, transplant recipients have a 30-80-fold higher risk of developing NMSC [38]. 

It has been reported that several class I and class II HLA allele groups were associated with SCC in immunosuppressed patients, including HLA-B*27, HLA-A*03 and HLA-DQA1*01 [31]. However, other authors did not confirm these association [26,39]. Indeed, Glover et al. no longer observed the previously-reported negative association between HLA-A*11 and SCC [39]. In addition, it has been reported that multiple studies found no association between HLA-DRB1/01 and SCC in immunosuppressed patients, in contrast to the positive association reported in immunocompetent patients [31]. 

Associations in renal transplant patients were not confirmed by Bavnick et al., describing conversely a reversal of association between HLA-A/11 and SCC, in contrast to multiple previous studies [40]. It has been postulated that these inconsistent conclusions may result from differing environmental factors affecting renal transplant patients in different countries [31]. Indeed, Yesantharao et al. proposed that HPV infection may have been a cofactor in SCC pathogenesis in the Dutch population [31,41], while the Australian ones were exposed to a greater excess of UV radiation as compared to the Dutch patients [40], thus neoantigens produced by UVR-induced DNA mutations may have played a much larger role in the etiology of SCC in these patients. In conclusion, these underlying differences in tumor antigens may have altered the association between SCC and HLA-A*11 in these population [31].

It has been postulated that the heterogeneous expression of class I HLA proteins in SCC may also explain why immunosuppression increases the SCC risk 65-fold, but BCC risk only 10-fold [42]. Indeed, immunosurveillance may better control the SCC pathogenesis because of the partial expression of class I HLA proteins in SCC, in comparison to BCC, where class I HLA proteins are often completely absent [43]. Consequently, a loss of immunosurveillance due to immunosuppression would influence SCC pathogenesis more than that of BCC.

Therefore, Yesantharao et al. proposed that the abnormal expression of the HLA-G protein on the surface of SCC cancer cells in immunosuppressed patients allowed for the evasion of immune surveillance [31]. The immunomodulatory effects of HLA-G under normal physiological conditions are well documented. Indeed, HLA-G in embryonic tissues, adult immune privileged organs, and in hematopoietic cells provide inhibitory signals to NK and T cells [42]. Consequently, HLA-G expression on SCC tumors could allow tumoral cells to negatively regulate NK and T lymphocyte-mediated destruction. In addition, it has been reported that HLA-G expression was present in various cancers (melanoma, breast, colon, lung and renal), and that melanoma cell lines expressing HLA-G isoforms had inhibited cytotoxic responses from NK and T-cells [44].

In addition, UV radiation also has suppressive effects on skin immunity. Indeed, it has been reported that UV radiation-induced photolesions such as cyclobutane pyrimidine dimers (CPDs) are immune-suppressive [45]. Furthermore, UV radiation also stimulates other molecules with immunosuppressive properties such as IL-10, prostaglandins, platelet-activating factor and ROS [46]. Moreover, UV radiation also inhibits mast cells, cytotoxic T cells and memory T cells, whereas regulatory B lymphocytes, T lymphocytes and natural killer cells are activated by UV radiation [45]. In addition, UV radiation also affects Langerhans cells (LC), reducing LC numbers in the skin by inducing LC migration to the draining lymph node [47]. All these findings highlight sharply the clear relationship between UV radiation and immunosuppression.

## 3. AK and SCC Genetic Profile

AKs are the most frequent precancerous lesion in humans; they develop usually in fair-skinned people on sun-exposed areas [48,49]. Between 0.025% and 16% of AKs evolve to iSCC every year [45,48]. Because every patient shows several AKs, the annual risk of developing iSCC has been reported as between 0.15 to 80% [49]. AK and iSCC have a similar genetic profiles, including alterations in the p53 gene [45]. Pathologically, these alterations are described as hyper-chromatic and pleomorphic nuclei with alteration of the nuclear cytoplasmic ratio, loss of polarity, and cellular superposition [49]. Cytological atypia at the basal layer of the AK can determine progression to SCC. Nevertheless, not all AKs show this behavior. Indeed, many AKs persist in the same stage, while others will regress and a few will progress into iSCC [49]. It has been reported that the risk of progression varies up to 16% and the evolution of a particular lesion is unpredictable [50]. AK is the most common precursor of cutaneous iSCC, and it represents a disease continuum [2]. AK can remain stable, regress, relapse or progress [2]. Every AK starts with an atypical basal layer [2,4,48]. It has been reported that the progression from AK to iSCC of the skin follows a pathway similar to the ones of cervical cancer [2,48]. However, cutaneous iSCC could also develop from atypical basaloid localized at the epidermal basal layer (AK I) [2,4]. This pathway, known as the differentiated pathway, is the most frequent way that leads to cutaneous iSCC [2,4,48]. However, the progression from AK I to AK II and AK III (classic pathway) has been described in several iSCC cases [2]. Therefore, it could be concluded that all AK lesions are potentially invasive [2]. The cell will loss polarity, polygonal shape and cell-cell contacts, and will switch from epithelial to mesenchymal type, with a significant difference in E-cadherin, β-catherin, vimentin and Ki67, without significant differences in podoplanin (D2-40), p16 and p53 [2,4]. Proliferative AK is a new subtype of AK that exhibits proliferative characteristics both histologically and clinically [2,48]. Proliferative AK is resistant to standard therapies because of deep migration of abnormal cells along hair follicles and sweat ducts. It has a strong propensity to develop infiltrative SCC and may occur concomitantly with BCC [2,48].

However, it has been also observed that AKs are unlikely to be precursors of SCCs in the Japanese population. Indeed, in a study on Japanese populations, loss of heterozygosis (LOH) was detected in seven of 37 AKs analyzed, but in only one of 14 SCCs evaluated [51]. In addition, microsatellite instability was not detected in all the AK or SCC analyzed specimens [51].

Mitochondrial DNA (mtDNA) is known to be subject to the loss of a significant proportion of specific sections of genetic code due to exposure to UV radiation and aging [52]. It has been demonstrated in several studies that the mtDNA4977 and mtDNA3895 deletions are more frequent in sun exposed areas. In particular, it has been reported that mtDNA4977 deletion could be an indicator of developing NMSC. Indeed, Powers et al. found that mtDNA deletions were related to sun exposure [53]. In the same study, Powers et al. demonstrated that SCC which had arisen in non sun-exposed skin had a percentage of mtDNA4977 that was on average over three times the level observed in non sun-exposed skin in any other cohort [53]. This high percentage could be an indicator of predisposition to SCC development. Exposed skin showed elevated levels of the deletion. In addition, mtDNA4977 was present at a much higher level in perilesional skin. Follicular extension in AK, too, has a prognostic significance [49]. Infundibular, isthmic, and sub-isthmic atypia have been reported in 25%, 63.6%, 100%, respectively, of iSCC [49]. These findings have implications for identifying patient factors which would be predictive of the progression of actinic keratosis to invasive carcinoma, providing potentially valuable patient screening guidelines. Stem cell quiescence acts as a tumor suppressor in squamous tumors [49].

### 3.1. SCC Proliferation 

SCC is the second most frequent type of NMSC, with over 250,000 new cases per year in the US [1]. Several risk factors have been reported for SCC development, including aging, fair complexion, chronic skin ulcers, burn scars, immune suppression, and exposure to ultraviolet light and chemical carcinogens [2]. SCCs usually have favorable prognosis, but around 4% of the patients develop metastases and 1.5% eventually die [2]. SCC behavior is characterized by possible progression from a AK precursor to SCC in situ, iSCC, and finally metastatic SCC [54]. The evolution from AK to iSCC occurs following progressive stages of keratinocyte intraepidermal neoplasia (KIN). Based on these stages, AKs with atypical keratinocytes (KIN I) could progress to lesions with atypical keratinocytes in the lower two-thirds of the epidermis (KIN II) and then to lesions with full thickness epidermal neoplasia (KIN III) [54]. In a recent study, Fernandez-Figueras reported that AK I, AK II and AK III lesions overlying invasive SCC were present in 63.8%, 17.9% and 18.4% of cases, respectively [54]. In addition, the authors showed that AKs I, AKs II and AKs III were detected in 77.9%, 6.6% and 8.3% of cases respectively [54]. These results indicate that direct transformation from AK I to iSCC (the so-called “differentiated pathway”) is the most prevalent mechanism of transformation [54].

Complement factor H (CFH) is a soluble molecule that inhibits one of the three pathways that activates the complement C3, the alternate pathway. In SCC, it supports the proliferation and migration of malignant cells. Indeed, it has been reported that the progresses from AK to SCC is characterized by a high expression of CFH, factor H-like protein-1 (FHL-1), and CF-1—three important markers of inflammation [55]. Therefore, a high CFH expression in SCC is associated with negative prognosis [55]. Furthermore, it has been highlighted that SCC cells produce CFH, escaping the complement mediated cell destruction, which leads to faster SCC progression [55]. Another protein that stimulates SCC malignant cell proliferation is Serpin A1 [56], that is overexpressed in SCC in comparison to normal keratinocytes [57]. In addition, the Serpin A1 level is related to SCC invasiveness [57]. Moreover, Serpin A1 level are increased by TNF-α, IFN-γ and IL-1β, showing the relationship between inflammation and tumorigenesis [57].

### 3.2. Abnormal Cell Surface Expression of HLA Protein in SCC 

Downregulation of the HLA-I protein expression reduces the presentation of tumor antigens to CD8+ T lymphocytes, diminishing T lymphocyte mediated destruction of SCC cells [58]. Multiple studies suggest a role for immunoselection in carcinogenesis via HLA-I protein expression alteration [31]. Selective downregulation would allow SCC cells to escape recognition by CD8+ T cells. In addition, the overall decrement in HLA-I proteins on the cell surface may increase the ability of SCC cells to escape immune surveillance [31].

### 3.3. Alteration of APC Gene

LOH of the adenomatous polyposis coli (APC) gene has been reported in several neoplasia, including SCC [8]. APC plays a pivotal role in microtubule assembly and leads to β-catenin destruction, which activates the transcription of oncogenes, such as Myc and Cyclin D1. It has been reported that in SCC samples, APC was expressed only in the nucleus of proliferating cells [59]. Conversely, in normal skin APC was detected only in the cytoplasm [59]. However, APC was also found in the nucleus of cells that surrounded SCC, suggesting that these could be exposed to the genetic changes that modified the normal APC expression [59].

## 4. BCC Risk Factors

BCCs arise from basal cells, which are a layer of cells located at the deepest part of epidermis. Basal cells have recently come to be considered skin stem cells, as they are constantly proliferating and generating keratinocytes, which are continuously pushed to the surface and eventually become a dead layer of stratum corneum. In developing BCC, several risk factors are involved, including Fitzpatrick phototype I and II, sunburns in childhood, family history of skin cancer, immunosuppression, high cumulative UV exposure, and exposure to carcinogenic chemicals, especially arsenic [60]. Among them, UV radiation is thought to be the most important risk factor. Indeed, 80% of BCC arises on sun-exposed areas, especially the head and neck [54]. Regional differences, including hair follicles density, could clarify why the dorsal area of the hands is usually not involved by BCC, despite extensive sun exposure [61]. Contrarily to SCC, BCC is believed to arise de novo [54]. In a recent study, Powers et al. reported that BCC had three times more genomes bearing mtDNA4977 in sun exposed skin compared to non sun-exposed skin [53]. As is already known, the mtDNA4977 has proved to be an indicator of possible risk of developing NMSC [53]. The high percentage of deletion bearing genomes in skin described as non sun-exposed may be due to poorly recalled exposure of the selected area. In addition, perilesional skin had an equal percentage of deletion, suggesting that exposed but still normal skin had endured levels of exposure similar to skin in proximity to a lesion [53].

### 4.1. Sporadic BCC 

Sporadic BCC is characterized by several genetic alterations, which affected mainly the sonic hedgehog (SHH) patched (Ptch) 1 signaling pathway [62]. The SHH pathway plays a pivotal role in embryonic development [62]. Indeed, SHH signaling influences morphogenesis of the epidermis and its appendages [63]. Although most of BCCs are sporadic, several cases are caused by basal cell nevus syndrome (BCNS), an inherited disorder characterized by multiple BCCs and other types of tumors [64]. BCNS patients show an inactivating mutation in the human homolog of the *Drosophila* gene Ptch, which regulates the SHH pathway [65]. This gene is also inactivated in sporadic BCCs [66]. Therefore, it could be concluded that Ptch1 normal function is required for the suppression of BCC.

Indeed, glioma 1 and 2 have been reported as altered in sporadic BCC [67]. FOXM1, a Forkhead box protein, has also been described as mutated in sporadic BCC, resulting in hyperproliferation of tumoral cells [68]. Recently, Asplund et al. have identified 201 upregulated and 160 downregulated genes in BCC cells compared to normal basal cells, including aquaporin 3, envoplakin, desmoglein 2, and MHC class II proteins [68]. In BCC pathogenesis, several immune-related markers have been reported [69]. On the one hand, the BCC inflammatory infiltrate is principally influenced by Th2 cytokines, linked to immunosuppression. On the other hand, regressing BCC are modulated by Th1 cytokines, especially by interferon (IFN)-γ that functions as a tumor suppressor [69]. IL-17, IL-23 and IL-22 play a pivotal role in inflammatory diseases of the skin, but their role in skin carcinogenesis is not completely understood [69]. However, it has been found that IL-17 is produced by both CD4+ and CD8+ T cells and it is related to IFN-γ secretion [56]. 

### 4.2. Recurrent BCC

In recurrent BCC, several risk factors play an important role, such as topography (centrofacial and periauricular region), diameter of the lesion, and age > 60 years [60]. It has also been demonstrated that in recurrent BCC, cyclooxygenase-2 (COX-2) was overexpressed. Indeed, over 90% of recurrent BCC expressed COX-2 compared to only 59.1% of sporadic BCC [70]. In addition, the overexpression of COX-2 was related to increased levels of vascular endothelial growth factor-A and regulators of apoptosis, such as Mcl-1 and Bcl-2 [70]. Matrix metallopeptidase 9 (MMP-9) has also been reported as overexpressed in BCC [71]. MMP-9 plays a role in neutrophil migration across the basement membrane, angiogenesis, and neovascularization, and in collagen contraction [71]. Indeed, MMP-9 was demonstrated to infiltrate BCC by in situ hybridization in the stromal fibroblasts around the tumor and squamous cell carcinomas, and was found in the reactive eosinophils infiltrating the dermis [70].

## 5. Role of Keratinocytes-Specific Proteins

The process of skin carcinogenesis is still not fully understood. However, several studies have been conducted to better explain the mechanisms that lead to malignancy. More than 50 keratinocytes-specific proteins have been described by Paulitschke et al. [5]. On one hand, several of these, such as IF regulatory factor 6 and alpha-2 macroglobulin-like protein 2, play a pivotal role in keratinocyte proliferation and differentiation. On the other hand, other proteins, such as calmodulin-like protein 5, are involved in keratinocyte differentiation. It has been reported that IL-1 beta could modulate the production of keratinocyte proteins in inflammation, leading to a reduction in the expression of both keratinocyte differentiation and motility proteins [5]. Otherwise, IL-1 also affects the synthesis of angiogenetic and anti-apoptotic proteins, leading to a higher expression of both [5]. Therefore, it has been postulated that IL-1 could play a pivotal role in skin carcinogenesis.

## 6. Role of ROS and NO

AK shows the earliest changes at the basal layer of the interfollicular epidermis [49]. Indeed, inactivation of p53 induced by UVB has been demonstrated in the basal keratinocytes of AK [49]. In addition to direct DNA damage, UV damage leads to the production of ROS and reactive nitrogen intermediates, which also cause oxidative damage to DNA. Furthermore, both UVB and UVA have been demonstrated to increase the level of cutaneous nitric oxide (NO) by inducing NO synthase [72]. As a consequence, high NO and high ROS levels lead to peroxynitrite production, formed by the combination of NO and ROS. Peroxynitrite is extreme toxic to DNA [73].

## 7. Role of Angiogenesis

Angiogenesis is the formation of new blood vessels from pre-existing ones and this process has an important role in tumor formation, invasion, and metastatization [74]. Vascular endothelial growth factor (VEGF) is the most important factor in angiogenesis [75]. On one hand, VEGF promotes intracellular transduction pathways. On the other hand, VEGF supplies the new vessels with nutrients and oxygen, leading to more aggressive tumor behavior and metastatization [76]. VEGF single-nucleotide polymorphisms (SNPs) can be detected in regulatory regions, affecting VEGF expression or activity [77,78]. VEGF polymorphisms have been previously reported in several carcinomas, including esophageal squamous cell carcinoma, and oral squamous cell carcinoma [78,79,80]. The VEGF gene −460 C>T polymorphism and −1154 G>A polymorphism have been reported as possible markers of prognosis in SCC [79].

## 8. Animal Models to Study in NMSC

Classical mouse models do not produce neoplasia of the BCC lineage [81]. Nowadays, several mouse models in which SHH signaling could be manipulated have been developed with the aim of studying BCC in vivo [81]. Thanks to these models, chemoprevention and chemotherapy, as well as BCC pathogenesis, have been studied. Indeed, it has been found that SHH signaling plays a pivotal role in BCC carcinogenesis. In addition, it has been shown that the overexpression of the Gli family of transcription factors (GLI) 1 or GLI 2 can lead to BCC-like proliferations [82]. Furthermore, BCC arises in Ptch1+/− mice, often showing the deletion of the wild-type copy of Ptch1 and the upregulation of SHH signaling [83]. Thus, it can be concluded that mouse models with mutations in the genes that encode four or more different parts of SHH signaling develop at least BCC-like neoplasia. In addition, it has been reported that the higher the activation of SHH signaling, the more the neoplasia resemble human BCC, while with lower SHH activation the tumors are more hair-follicle-like [83].

It has also been shown that p53 loss strongly increases tumorigenesis via the SHH pathway [81]. Indeed, Ptch1+/− mice in which p53 is deleted have shown a strong increase of BCC formation [81]. Therefore, it has been postulated that the high incidence of p53 mutations in human BCC is probably not caused by UV radiation, but rather reflects the fact that p53 loss is involved in the development of BCC [81].

Mice treated with UV, IR, or with chemical carcinogens develop SCC [81]. Indeed, it has been demonstrated that p53 increases in epidermal cells after exposure to UV radiation in hairless SKH-hr1 mice [84]. It has also been reported that p63 −/− mice keratinocytes show a reduced proliferative capability, probably because of increased expression of p21, a direct transcriptional repression target of p63 [85]. P63 affect Notch activation, regulating the balance between keratinocyte self-renewal and differentiation. A microarray analysis for p63 target genes in genetically complemented mice has also shown that the function of p63 in epithelial development was partly mediated by IkB kinase-alpha and GATA-3 [85]. It has also been reported that Fas ligand-deficient mice develop more p53 mutations than wild-type mice after prolonged UV irradiation [86]. Indeed, after a transitory upregulation of Fas and Fas ligand expression induced by acute exposure to UV, a reduction of Fas ligand expression has been observed after 1 week of continuous UV irradiation in mice, leading to a decrease in the number of apoptotic cells [86]. Finally, it has been demonstrated that transfected NIH 3T3 cells induce tumors at the subcutaneous site of injection and spontaneous lung metastases in nude mice [87]. 

## 9. Conclusions

Non-melanoma skin cancers (NMSCs) are the most common malignancy worldwide, of which 99% are basal cell carcinomas (BCCs) and squamous cell carcinomas (SCCs) of skin. NMSCs are relatively non-lethal and curable by surgery, hence are not reportable in most cancer registries around the world, yet they currently pose an increasing global healthcare problem due to rising incidence. Both basal cells and squamous cells belong to keratinocytes, therefore sometimes BCC and SCC are termed keratinocyte cancer. These three types of cancer share many characteristics, yet they are very different from etiology to progression. One shared characteristic of skin cancer is that, according to the current views, they all are caused by solar or artificial ultraviolet radiation (UVR). UVA and UVB from solar UVR are the major UV bands reaching the earth surface. Both UV types cause DNA damage and immune suppression, which play crucial roles in skin carcinogenesis. UVB can be directly absorbed by DNA molecules and thus causes UV-signature DNA damages. UVA, on the other hand, may function through inducing cellular ROS which then causes oxidative DNA damages. Although skin carcinogenesis is still not fully understood, several papers have demonstrated the presence of genetic and molecular alterations involved in this process. In addition, plenty of NMSC risk factors are now known, allowing for an effective prevention of NMSC development, especially in elderly people. This has led to a shift in emphasis on prevention of NMSCs with the development of various skin cancer prevention programs worldwide. Improving knowledge on skin cancer pathogenesis will improve NMSC management with a focus on prevention, screening, diagnosis, and staging. As reported, avoiding excessive exposure to UV radiation, particularly prolonged or midday sunlight exposure, and use of sun-protective clothing and sunscreens is the most important factor in preventing NMSC formation, since cancer field can be the first step of invasive SCC development. However, this issue is complicated by a lack of knowledge on the amount of sun exposure required to cause skin cancer. More studies are needed in this field.
